# Fusing Prediction and Perception: Adaptive Kalman Filter-Driven Respiratory Gating for MR Surgical Navigation

**DOI:** 10.3390/s26020405

**Published:** 2026-01-08

**Authors:** Haoliang Li, Shuyi Wang, Jingyi Hu, Tao Zhang, Yueyang Zhong

**Affiliations:** School of Health Science and Engineering, University of Shanghai for Science and Technology, Shanghai 200093, China

**Keywords:** mixed reality, respiratory motion compensation, adaptive kalman filter, respiratory gating

## Abstract

Background: Respiratory-induced target displacement remains a major challenge for achieving accurate and safe augmented-reality-guided thoracoabdominal percutaneous puncture. Existing approaches often suffer from system latency, dependence on intraoperative imaging, or the absence of intelligent timing assistance; Methods: We developed a mixed-reality (MR) surgical navigation system that incorporates Adaptive Kalman-filter-based respiratory prediction module and visual gating cues. The system was evaluated using a dynamic respiratory motion simulation platform. The Kalman filter performs real-time state estimation and short-term prediction of optically tracked respiratory motion, enabling simultaneous compensation for MR model drift and forecasting of the end-inhalation window to trigger visual guidance; Results: Compared with the uncompensated condition, the proposed system reduced dynamic registration error from (3.15 ± 1.23) mm to (2.11 ± 0.58) mm (*p* < 0.001). Moreover, the predicted guidance window occurred approximately 142 ms in advance with >92% accuracy, providing preparation time for needle insertion; Conclusions: The integrated MR system effectively suppresses respiratory-induced model drift and offers intelligent timing guidance for puncture execution.

## 1. Introduction

Lung cancer remains the most commonly diagnosed cancer and the leading cause of cancer-related death worldwide. According to the Global Cancer Statistics 2022 (GLOBOCAN), there were approximately 2.5 million new cases and 1.8 million deaths, accounting for 12.4% of all cancer diagnoses and 18.7% of total cancer mortality [1].

Current percutaneous lung biopsy procedures primarily rely on two-dimensional image guidance, which suffers from limitations such as delayed intraoperative information updates and insufficient localization accuracy. This technique often requires repeated intraoperative CT scans to confirm the puncture path, not only prolonging surgery duration and increasing the risk of complications like bleeding and pneumothorax, but also leading to heightened radiation exposure for both medical staff and patients [2,3]. Furthermore, lung tissue displacement caused by respiratory motion presents another critical challenge to precise puncture. Studies indicate that during a single respiratory cycle, the displacement of the target lung region can reach up to 2 cm, significantly increasing positioning errors and surgical difficulty [4].

Driven by the evolving concept of precision medicine and rapid advances in Augmented Reality (AR) and Mixed Reality (MR) technologies, AR/MR surgical navigation systems are emerging as pivotal tools in modern minimally invasive surgery [5,6]. By precisely registering and overlaying three-dimensional virtual models reconstructed from preoperative medical images onto the patient’s actual anatomical structures within the surgical field, these systems provide surgeons with intuitive “X-ray vision” capabilities. This technology holds the potential to revolutionize surgical visualization and positioning accuracy [7,8,9].

Early research primarily focused on preoperative navigation, utilizing three-dimensional reconstruction of CT or MRI imaging data to provide surgeons with clear visualizations of pulmonary anatomical structures. For instance, Leonardo Frajhof et al. applied augmented reality and 3D printing technology to preoperative planning for pulmonary lesions. Printed models assisted surgeons in understanding patient anatomy and spatial relationships, thereby supporting surgical pathway planning [10]. However, this approach has significant limitations, being confined to preoperative static models and unable to support the dynamic changes in anatomical structures during surgery. To address the limitations of preoperative navigation, research has increasingly shifted toward intraoperative navigation. By overlaying three-dimensional models onto the surgical field, MR and AR technologies provide surgeons with dynamic guidance during actual procedures. For instance, Chengrun Li et al. successfully applied augmented reality technology in lung cancer surgery, delivering intuitive intraoperative guidance [11].

However, the periodic deformation and displacement of thoracic and abdominal organs caused by respiratory motion pose a significant challenge to the stability and reliability of MR navigation. During respiration, the MR overlay model derived from static registration continuously deviates from the actual organ positions. This error not only misleads physicians but may also directly lead to puncture failure or complications [12,13]. To address this challenge in current clinical practice, breath-hold puncture is commonly employed. While effective, this strategy heavily relies on the physician’s experience and the patient’s perfect cooperation [14].

In recent years, numerous researchers have focused on addressing respiratory motion issues through technological means. Some studies employ intraoperative CT [15] or MRI [16] for image registration and updating, but this approach inevitably exposes both medical staff and patients to ionizing radiation.

Another category of research explores compensation methods based on models correlating external respiratory signals with internal organ motion [17]. Among these, Kalman filters and their variants are widely applied for respiratory motion tracking and prediction—particularly in radiation therapy—due to their ability to achieve optimal estimation in noisy environments [18].

This paper designs and implements a mixed reality surgical navigation system integrating Adaptive Kalman filter prediction with intelligent gate-controlled prompts. The core innovation lies in establishing an intelligent closed loop from physiological signal sensing to MR visualization feedback: the Adaptive Kalman filter predicts respiratory states hundreds of milliseconds ahead, proactively compensating for inherent MR display latency to stabilize virtual models while precisely anticipating the safe window at the end of inspiration. This enables proactive MR-based puncture alerts for surgeons. Furthermore, to objectively validate system performance, we independently developed a dynamic respiratory motion simulation platform, providing a high-fidelity, quantifiable experimental environment for evaluating algorithms and systems.

## 2. Materials and Methods

**Figure 1:**
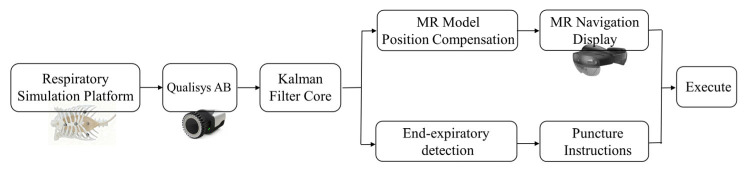
The main system framework.

To address the challenges posed by respiratory motion during mixed reality-guided puncture procedures, we developed and validated an integrated experimental platform. The platform employs a workflow design encompassing three key components: data processing, motion prediction, and visual guidance. As shown in Figure 1, the core components of this system primarily include: (1) a head-mounted display device (Microsoft HoloLens 2, Microsoft Corp., Redmond, WA, USA) equipped with a mixed reality navigation application, (2) a high-precision optical tracking system (Qualisys AB, Gothenburg, Sweden), capable of sub-millimeter-level positioning, using Qualisys Track Manager (QTM, version 2022.3), and (3) a simulation platform for mimicking human respiratory movements (Respiratory Motion Simulation Platform, supporting multi-mode waveform simulation). The synergy between these components enables the real-time tracking, prediction, and compensation of respiratory motion.

### 2.1. System Architecture Design

**Figure 2:**
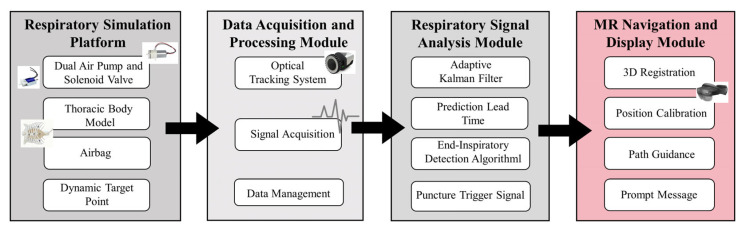
Overall System Architecture. Arrows indicate the data flow between system modules.

The mixed reality surgical navigation system developed in this study aims to address the challenges of dynamic registration and precise puncture guidance under respiratory motion interference. As shown in Figure 2, the system constitutes an integrated architecture encompassing perception, decision-making, and feedback, primarily comprising four core modules.

Respiratory Motion Simulation Platform: This module serves as the validation benchmark for this study. It consists of a transparent thoracic manikin, an internal silicone airbag, and a precision pneumatic control system based on dual air pumps and a three-way solenoid valve. It generates known and controllable dynamic thoracic displacement according to preset respiratory waveforms, simulating physiological respiratory motion.

Data Acquisition and Processing Module: This module serves as the system’s “sensory” component. A high-precision optical motion capture system continuously tracks passive optical markers affixed to the surface of a dynamic manikin in real time. The resulting respiratory displacement signals function as the spatiotemporal reference for the entire system and as input observations for the Adaptive Kalman filter. The commercial high-precision optical motion capture system Qualisys motion capture system (Qualisys AB, Gothenburg, Sweden) is employed, equipped with three infrared cameras operating at a sampling frequency of 100 Hz.

Prediction and Compensation Core Module: This module serves as the core of the system and is key to achieving dynamic accuracy enhancement. We designed and implemented adaptive Kalman filter for real-time state estimation and short-term prediction of respiratory motion. By integrating the system’s dynamic model with observational data from the optical tracking system, this filter outputs optimal estimates of future respiratory states (including position and velocity) over the next several hundred milliseconds. These predictions serve dual purposes: (a) Compensating for inherent delays in MR displays to stabilize virtual models; (b) Precisely forecasting impending end-inspiratory plateaus to inform decision-making for puncture gating.

Mixed Reality Navigation and Visual Interaction Module: This module serves as the interface between the system and the surgeon. Microsoft Hololens2 is responsible for presenting navigation information. Its core functions include: (a) loading and rendering a 3D lung model reconstructed from preoperative CT data; (b) receiving predictive data from the Adaptive Kalman filter core to dynamically update the spatial pose of the virtual model, synchronizing it with compensated physiological motion; (c) Triggering a clear, high-contrast visual cue within the surgeon’s actual surgical field when predicting the end-inspiratory window, thereby enabling intelligent guidance for optimal surgical timing.

### 2.2. Implementation of the Adaptive Kalman Filter Algorithm

**Figure 3:**
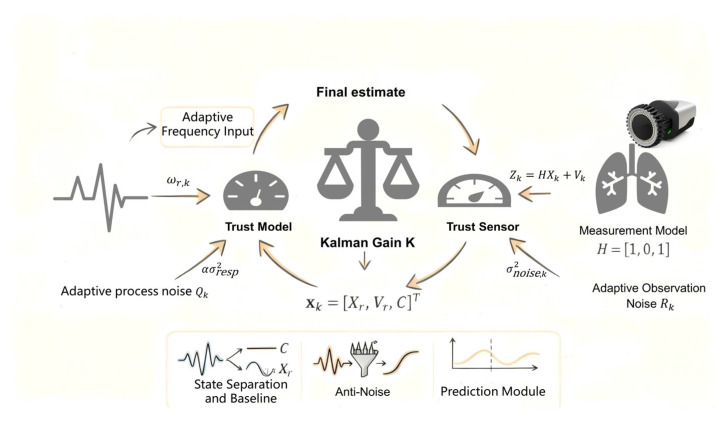
Algorithm Workflow Diagram.

To overcome system latency and achieve predictive navigation, we designed and implemented Adaptive Kalman filter-based prediction framework for estimating respiratory motion states and compensating for the resulting dynamic errors. The complete workflow of this algorithm is illustrated in Figure 3.

#### 2.2.1. State Space Model

The respiratory motion is modeled using a three-dimensional state vector, as shown in Formula (1):

(1) $$x_{k}=\left[\left.\begin{array}{c} X_{r}\\ V_{r}\\ C \end{array}\right]\right.,$$

where $X_{r}$ is the respiratory displacement, $V_{r}$ is its velocity, and C is the slowly varying baseline offset. The state prediction from step k−1 to k is given by Formulas (2)–(4):

(2) $$x_{k}^{-}=A_{k}x_{k-1},$$

(3) $$A_{k}=\left[\left.\begin{array}{ccc} 1 & \Delta t & 0\\ -\omega_{r,k}^{2}\Delta t & 1 & 0\\ 0 & 0 & 1 \end{array}\right]\right.,$$

Here,

(4) $$\omega_{r,k}=2\pi f_{r,k},$$

denotes the instantaneous angular breathing frequency. This representation effectively encodes the oscillatory respiratory dynamics.

The observation model describes the relationship between the state and the measured values, as shown in Formulas (5) and (6). In this system, the observation $Z_{k}$ is derived from the displacement measured by the optical tracking system, and its observation equation is:

(5) $$Z_{k}=HX_{k}+V_{k},$$

(6) $$H=[\begin{array}{ccc} 1 & 0 & 1 \end{array}],$$

where H denotes the observation matrix, and $\nu_{k}$ represents the observation noise, which follows a zero-mean Gaussian distribution $\nu_{k}∼N(0,R)$.

This observation model reflects the physical measurement mechanism of the optical tracking system: the respiratory displacement $X_{r,k}\;$ contributes directly to the measured signal, the baseline offset $C_{k}$ appears as an additive bias, and the respiratory velocity $V_{r,k}$ is not directly observable. Therefore, the coefficients in H are determined by the measurement physics rather than empirical tuning.

#### 2.2.2. Adaptive Estimation

Respiratory frequency $f_{r,k}$ is estimated from a 20 s buffer of the filtered signal using peak detection and smoothed using a 0.05 Hz IIR low-pass filter. The updated frequency directly modifies $A_{k}$, forming the closed-loop adaptation mechanism. $R_{k}$ as shown in Formula (7).

(7) $$R_{k}=\sigma_{\mathrm{noise},k}^{2},$$

where $\sigma_{\mathrm{noise},k}$ is obtained from the high-frequency components of the raw measurement via numerical differentiation. $Q_{k}$ as shown in Formula (8).

(8) $$Q_{k}=\left[\left.\begin{array}{ccc} 1 & 0 & 0\\ 0 & \omega_{r,k}^{2} & 0\\ 0 & 0 & \alpha \sigma_{\mathrm{resp}}^{2} \end{array}\right]\right.\;,$$

with $\sigma_{\mathrm{resp}}^{2}$ computed from a long-term buffer of $X_{r}$ and α=0.01.

To generate predictions over a longer time horizon, iterative propagation of the state model is performed, as shown in Formulas (9) and (10):

(9) $${\hat{x}}_{k+h|k}=A_{k}^{h}x_{k},h=1,2,\ldots ,H,$$

The predicted respiratory signal is therefore:

(10) $${\hat{X}}_{r,k+h|k}=[\begin{array}{ccc} 1 & 0 & 0 \end{array}]A_{k}^{h}x_{k},$$

This model-based propagation leverages: time-varying frequency $\omega_{r,k}$, dynamic process noise $Q_{k}$, oscillatory nature of the respiratory system. The prediction horizon h determines the temporal lead of the generated prompt, with larger h yielding earlier but potentially less accurate phase estimates. Because $A_{k}$ is continually adapted from real-time frequency estimates, the multi-step prediction remains well-aligned with the current breathing phase and amplitude.

Liu et al. compared three methods: end-inspiratory breath-hold, random breath-hold, and end-expiratory breath-hold. Results showed 100% puncture success rates across all three groups, but the end-expiratory breath-hold group had the highest complication rate (58.2%) [19]. This indicates that while end-expiratory breath-hold is feasible, it demands high patient cooperation, and repeated breath-holds prolong procedure duration. Therefore, this system selects the end-inspiratory phase as the respiratory trigger.

The system identifies the end-inspiration moment in real time by analyzing the velocity and displacement signals output from the filter. Its decision logic is as follows: When the velocity signal crosses zero from positive to negative, and the predicted displacement is at the end of an upward trend, when these conditions are met, the system determines that the end-inspiration phase is imminent and triggers the MR visual alert in advance, providing the surgeon with sufficient reaction time. To enhance the robustness of respiratory signal monitoring and prevent false alarms caused by motion artifacts or transient physiological variations, a historical data-based adaptive validation module was incorporated into the core detection algorithm. This module dynamically constructs a personalized reference respiratory template through online learning of recent stable breathing patterns, thereby establishing an adaptive threshold range. Each real-time detected respiratory event must subsequently pass a dual-validation process: it is first assessed by the primary algorithm and then evaluated for its similarity to the learned template. An event is only confirmed as a valid physiological signal when both checks are consistent. This redundant design significantly improves the system’s ability to discriminate abnormal signals and enhances its clinical reliability.

**Figure 4:**
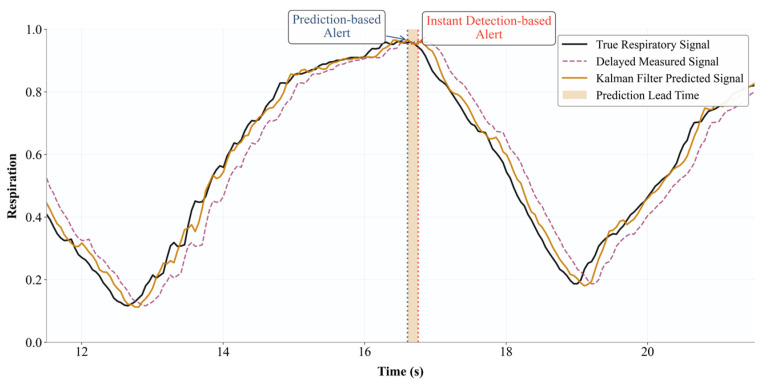
Schematic Diagram of Adaptive Kalman Filtering Task.

As shown in Figure 4, the delayed measurement signal lags behind the actual respiratory signal, while the signal filtered through the Adaptive Kalman filter closely follows the true curve.

### 2.3. MR Navigation System

Current marker-based registration methods require physical target attachment to patients, introducing clinical limitations including compromised sterility, registration drift due to tissue deformation, and workflow interruptions caused by manual re-registration. Furthermore, for multiple consecutive surgeries, each procedure necessitates re-marking QR codes and lesion locations, repackaging them into the HoloLens for accurate matching—a time-consuming process.

To overcome these limitations, this study establishes a markerless registration framework using a calibration plate. Its key innovation lies in automating CT-to-HoloLens coordinate transformation through a two-stage process: first locating targets in CT space, then tracking the plate in the MR environment. By acquiring marker coordinates in both spaces, registration is achieved.

This method employs a specially designed calibration plate as a common spatial reference carrier. A calibration plate with five non-coplanar QR codes was used for registration. While three non-coplanar markers are theoretically sufficient for rigid pose estimation, additional QR codes were included to improve robustness and reduce sensitivity to detection noise through overdetermined least-squares optimization. The core design of this calibration plate involves the fixed arrangement of five high-contrast QR codes at distinct positions. The spatial layout of these QR codes is meticulously engineered to ensure that any three points are non-collinear and all five points are non-coplanar. This uniquely defines a stable calibration plate coordinate system in three-dimensional space, effectively preventing singularity issues during subsequent spatial transformation solutions.

This study employs Microsoft HoloLens 2 and Vuforia detection capabilities for visual localization. This selection is based on the following rationale: First, the research focuses on validating the algorithmic workflow within a controlled environment, where absolute positioning accuracy is determined by the criterion of not obscuring motion signals. Research confirms that under such conditions, HoloLens 2 achieves millimeter-level positioning accuracy using QR codes [20], meeting the study’s requirements. Second, the QR code solution offers simple integration and high stability, facilitating rapid prototyping and enabling focus on developing core algorithms for respiration prediction.

**Figure 5:**
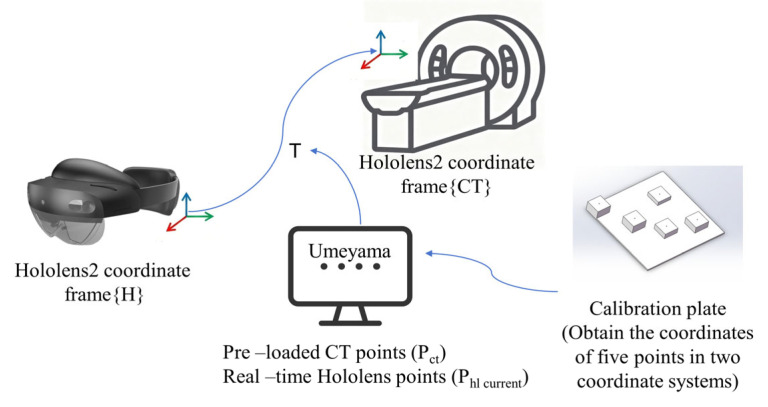
Markerless registration framework. Arrows indicate the data flow between system modules.

In this system, the CT coordinates are coordinates we created ourselves within our laboratory environment. The specific implementation process is as follows, as shown in Figure 5:

First, on the PC server, the precise 3D coordinates of the five QR code markers on the calibration plate within the CT coordinate system are preloaded and configured as foundational reference data for subsequent spatial registration. Subsequently, the server program is launched, opening the predefined Socket communication port to enter a listening state, ready to receive connection requests and data from the HoloLens client.

The HoloLens 2 client launches its MR application, utilizing the Vuforia engine to perform real-time recognition of the five QR codes on the calibration plate within its field of view. This process acquires the six-degree-of-freedom pose for each QR code in the HoloLens world coordinate system, comprising three-dimensional position coordinates and rotation quaternions. The client program extracts the positional coordinate information of these five QR codes and asynchronously sends real-time coordinate data packets to the PC server via the established socket connection.

Upon receiving real-time coordinates, the PC server immediately invokes the core algorithm: using the predefined CT coordinates and the received HoloLens coordinates as inputs, it executes the Umeyama algorithm based on least-squares optimization. This precisely calculates the optimal rigid transformation matrix T (comprising rotation matrix R and translation vector t) required to map points from the CT coordinate system to the current HoloLens world coordinate system. Successful determination of T establishes a stable mapping relationship between the CT space and the current HoloLens space.

Subsequently, when any model defined in the CT coordinate system (e.g., 3D organ models, tumor contours, or surgical pathways) is input on the PC server, the server applies transformation matrix T to convert its coordinates to the real-time HoloLens world coordinate system. The transformed coordinate data is then transmitted back to the HoloLens 2 client in real-time via Socket. Finally, HoloLens 2 precisely overlays and registers the received virtual model onto the corresponding real-world location visible to the user through the device, achieving high-precision visualization.

**Figure 6:**
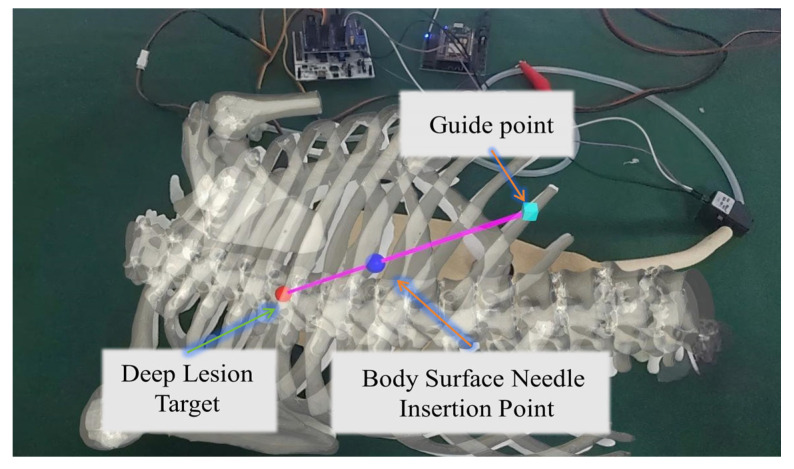
MR Display System.

Additionally, after completing registration, users can directly input and select multiple target points on the reconstructed 3D image. By selecting any two points (e.g., point A as the body surface needle insertion point and point B as the deep lesion target), the system will automatically generate a visual puncture path connecting the two points. As shown in Figure 6, the red dot represents the deep lesion target, while the blue dot indicates the body surface needle insertion point. A magenta path is generated between these two points. Furthermore, considering that during actual needle insertion, the needle’s internal position cannot be observed and the accuracy of the insertion angle cannot be confirmed, the needle path extends beyond the body surface needle insertion point. This allows users to externally adjust the needle insertion angle.

### 2.4. Breathing Simulation Platform

**Figure 7:**
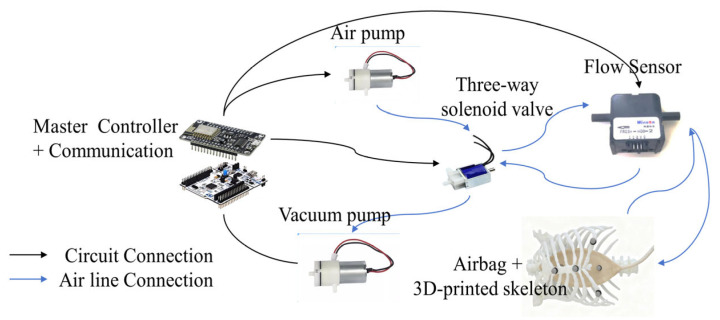
Breathing Simulation Platform.

The pneumatic control system employs two miniature diaphragm pumps (air pump maximum flow rate ~3 L/min, vacuum pump vacuum level ~−60 kPa) and a three-way solenoid valve (response time < 10 ms). The airbag is positioned within a 3D-printed transparent thoracic body model, with an initial volume of approximately 200 mL. As shown in Figure 7.

The core controller for this system utilizes the STM32 NUCLEO-F103RB development board, selected for its high performance, stability, and excellent expandability in embedded development. The STM32 NUCLEO-F103RB employs the STM32F103RBT6 microcontroller with an ARM Cortex-M3 core, operating at a maximum frequency of 72 MHz. It features 128 KB of Flash memory and 20 KB of SRAM, offering significant advantages in processing speed and storage capacity compared to traditional 8-bit microcontrollers. This provides ample performance support for executing complex control logic, processing sensor data, and managing TCP/UDP network communications (via an external ESP8266 module) in this project. Regarding hardware interfaces, the NUCLEO-F103RB offers 76 general-purpose I/O pins (some supporting PWM output and analog input), enabling flexible control of actuators like air pumps and solenoid valves while simultaneously acquiring signals from multiple sensors.

The pneumatic actuator unit serves as the core component for achieving respiratory simulation functionality, designed around the coordinated control of dual-pump drive and a three-way solenoid valve. After comprehensive evaluation, two miniature DC diaphragm air pumps operating at 5–6 V were selected, designated as the inspiratory and expiratory air sources, respectively. This pump type features low noise, low power consumption, and continuous operation, delivering more realistic airflow waveforms during alternating or simultaneous dual-pump operation. Paired with a normally closed three-way miniature solenoid valve for rapid switching between inhalation and exhalation air pathways, this valve offers short response times and a drive voltage matched to the system, ensuring dynamic simulation of the respiratory cycle (Appendix A).

As the lung-simulating reservoir, a highly elastic airbag is selected and positioned within a 3D-printed thoracic skeleton. The airbag is connected to the pneumatic system via silicone tubing, which provides both fixation and sealing through its inherent flexibility and excellent airtightness. This ensures no detachment or leakage occurs during repeated inflation and deflation cycles, thereby enabling stable simulation of the respiratory process.

The sensing and feedback unit of this system employs the FR03H series miniature flow sensor, integrated into the main gas pathway to enable real-time monitoring and closed-loop control of respiratory flow. Based on thermal mass flow measurement principles, the sensor features a D3 mm measurement bore and achieves a maximum flow rate of 5 L/min at 20 °C and 101.325 kPa. Its measurement accuracy is ±2.5% within the 0.15–5 L/min range, fully covering the typical flow range of simulated human tidal breathing. The sensor supports both I^2^C digital interface (100 kHz) and linear analog voltage output (0.5–4.5 V). It operates within a DC 4.9–14 V supply voltage range with typical operating current not exceeding 30 mA. Interface options include a PH2.0-5P plug-in connector or 2.54 mm-5P pins, facilitating seamless integration with STM32 controllers.

Sensors are installed in series within the main air pathway to ensure all inhaled and exhaled gases pass through their sensing elements, thereby obtaining accurate instantaneous flow measurements. The STM32 controller acquires this flow data in real time via I^2^C or analog channels and calculates tidal volume parameters by integrating the flow based on a preset algorithm. These values serve as feedback inputs for the PID control algorithm, dynamically adjusting the PWM duty cycle to precisely regulate the air pump speed and solenoid valve activation timing. Through this closed-loop control strategy, the system achieves high-fidelity tracking of target respiratory waveforms, significantly enhancing the realism and reliability of simulating various respiratory states.

### 2.5. Verification Methodology

**Figure 8:**
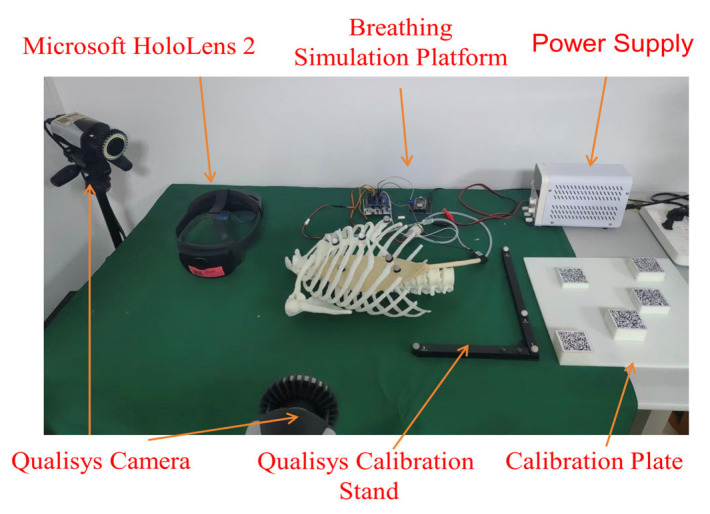
Experimental Platform.

To validate the performance of the integrated system proposed in this study, we constructed the complete experimental platform shown in Figure 8 and designed a series of quantitative and qualitative experiments. All experiments were conducted in a controlled laboratory environment to ensure the comparability and reproducibility of results.

We designed a systematic verification protocol aimed at quantitatively analyzing its dynamic registration accuracy, the precision and timeliness of respiratory gating prompts, and qualitatively assessing its clinical usability. To comprehensively evaluate system performance, we employed two typical respiratory waveforms for experimentation: (1) Standard sine wave: Serving as a benchmark, this simulates steady ideal respiration. Displacement amplitude was set to 10 mm with a 5 s period, corresponding to adult resting respiration. (2) Real-data-based respiratory wave: To assess the system’s robustness under non-ideal conditions, we employed real human respiratory waveforms sourced from public databases [21]. These waveforms encompassed Regular Breathing, Shallow Breathing, and Irregular Breathing. Following standardization, these waveforms matched the amplitude and period of the reference waveform.

When evaluating the impact of different breathing patterns on predictive model performance, this study analyzed respiratory signal data generated by a validated respiratory motion simulation platform. The platform’s preset waveforms were designed strictly within the clinically observed physiological range of human respiration to ensure physiological authenticity. Specifically, the waveform period references the typical respiratory rate range for normal adults at rest (10–20 breaths per minute, corresponding to a period of 3–6 s) [22]; baseline values for normal and shallow breathing amplitudes (10 mm and 3–5 mm) are established based on the AAPM TG-76 report [23], along with the irregular waveform range within the dataset.

The classification method builds upon the work of Jeong et al. [21], ultimately categorizing respiratory signals into three types: regular breathing, shallow breathing, and irregular breathing. The classification was based on three quantitative indices: the mean amplitude $M_{a}$, the amplitude irregularity $I_{a}$, and the phase irregularity $I_{p}$. The mean amplitude $M_{a}$ was defined as the average of the absolute amplitude of the respiratory signal S(t) , as shown in Formula (11):

(11) $$M_{a}=\frac{1}{N}\sum_{i=1}^{N}|S(t_{i})|,$$

which characterizes the overall breathing depth. The amplitude irregularity $I_{a}$ was calculated following the method proposed by Jeong et al. [21] and defined as the average of the standard deviations of all detected peaks $\left\{P_{k}\right\}$ and valleys $\left\{V_{l}\right\}$, as shown in Formula (12):

(12) $$I_{a}=\frac{\sigma (\{P_{k}\})+\sigma (\{V_{l}\})}{2},$$

The phase irregularity $I_{p}$ was used to quantify variability in the breathing rhythm and was computed as the average of the standard deviations of the respiratory cycle durations, namely the peak-to-peak intervals $\left\{T_{p,k}\right\}$ and the valley-to-valley intervals $\left\{T_{v,l}\right\}$, as shown in Formula (13):

(13) $$I_{p}=\frac{\sigma (\{T_{p,k}\})+\sigma (\{T_{v,l}\})}{2},$$

The latter two indices were used to evaluate fluctuations in breathing depth and rhythm, respectively. First, based on the distribution characteristics of the samples in the dataset, the median values of $I_{a}$ and $I_{p}$ were used as thresholds. Any respiratory signal for which either irregularity index exceeded the corresponding threshold was classified as irregular breathing. Subsequently, for signals with both irregularity indices below the thresholds, further discrimination was performed according to the mean amplitude $M_{a}$: signals whose $M_{a}$ fell within the lowest 25th percentile of all samples were defined as shallow breathing, while the remaining signals were classified as regular breathing.

#### 2.5.1. Three-Dimensional Registration Alignment Error Assessment

We used a high-precision optical motion capture system, the Qualisys motion capture system (Qualisys AB, Sweden), as the acquisition device for three-dimensional spatial coordinates. The system utilizes infrared optical tracking technology, whereby multiple synchronized Oqus cameras emit infrared light and capture the reflections from passive retro-reflective markers attached to the target object. Based on the principles of triangulation, the system reconstructs the three-dimensional position and attitude of the marker clusters in real-time. Spatial coordinate data were streamed and recorded via the Qualisys Track Manager (QTM) software. And the reflective markers were ensured to remain within the overlapping field of view of all cameras throughout the experiment.

According to manufacturer specifications and independent experimental evaluations, the Qualisys Oqus camera system achieves a static spatial accuracy better than 0.15 mm and a dynamic measurement error of approximately 0.26 mm when tracking objects in motion [24,25]. Given that the simulated respiratory motion in this study is characterized by low-frequency (typically below 0.5 Hz), smooth, and periodic displacement, the target velocities and accelerations remain well within the dynamic tracking capability of the optical system. Therefore, the measurement uncertainty introduced by the optical tracking system is negligible relative to the respiratory motion amplitude, and the system can be reliably regarded as a gold-standard reference for evaluating respiratory motion prediction and compensation performance.

First, set the recognition image to 5 cm × 5 cm within the Unity3D virtual scene (Unity Technologies, San Francisco, CA, USA; version 2021.3.23), matching the dimensions of the image registration environment. Place four small ball reference points on the model for error measurement, labeled 1–4. Once the 3D registration alignment is complete, the Micron Tacker probe is positioned at the virtual red sphere reference point and the edge of the real phantom. Each point is then tested at least 20 times, with the test results saved when the variance of their coordinates is within 0.2 mm. We define the Euclidean distance between the coordinates of the virtual red sphere reference point and the reference coordinates of the edge of the real phantom as the 3D registration alignment error (error), as shown in Formula (14). The experimental results are expressed as mean ± standard deviation.

(14) $$Error=\sqrt{(x_{1}-x_{2}{)}^{2}+(y_{1}-y_{2}{)}^{2}+(z_{1}-z_{2}{)}^{2}},$$

where x_1_, y_1_, z_1_ denote the virtual red sphere reference point coordinates; x_2_, y_2_, z_2_ denote the real phantom edge reference coordinates.

#### 2.5.2. Dynamic Quantified Registration Accuracy Evaluation

Dynamic registration accuracy is by comparing the deviation between the position of virtual target points displayed by the augmented reality system and the actual physical target point positions. The specific process is as follows:

First, select a specific target point within the lung nodule region of the thoracic phantom and define a corresponding homologous point on its virtual 3D model. Initiate the dynamic respiration simulation platform to operate according to the preset waveform. Simultaneously record the true 3D coordinates $P_{true}(t)$ of the target point provided by the optical motion capture system (serving as the “gold standard” for evaluation), along with the 3D coordinates $P_{ar}(t)$ of the homologous point calculated and displayed by the MR system through its registration algorithm.

Calculate the target point error at each time point throughout the entire data acquisition period, as shown in Formula (15):

(15) $$TPE(t)=||P_{true}(t)-P_{ar}(t)|{|}_{2},$$

Finally, we report the root mean square error (RMSE) and mean ± standard deviation of TPE(t) to comprehensively characterize the system’s dynamic tracking accuracy.

To quantitatively evaluate the contribution of the Adaptive Kalman filter prediction and compensation module, we established two primary comparison modes: (1) Mode A (Baseline Mode): Adaptive Kalman filter disabled. The MR system performs model updates and instantaneous end-inspiratory detection solely using real-time displacement measurements provided by the optical system, which include inherent latency. (2) Mode B: Enables the Adaptive Kalman filter. The MR system updates its model using displacement and velocity data that have undergone predictive compensation, triggering gated prompts based on the predictive results.

#### 2.5.3. Performance Evaluation of Respiratory Gating Prompts

This section evaluates the accuracy and timeliness of the system’s intelligent prompting functionality. Based on optical motion capture data, manually annotate the true time point $T_{gold}$ of the end-inspiratory phase within each respiratory cycle with precision. Record the time point $T_{prompt}$ at which the MR system triggers a visual prompt each time. Calculate the following metrics:

1. Prompt Accuracy: The percentage of total respiratory cycles in which the system correctly triggers a prompt ($T_{prompt}$ falls within a clinically acceptable tolerance window centered on $T_{gold}$).
2. Prompt Delay/Advance: Calculate $\Delta T=T_{prompt}-T_{gold}$. A negative ΔT value indicates the prompt was issued before the true end-inspiratory point, which is the desired effect of the prediction algorithm. We report the mean and standard deviation of ΔT, which reflects the effective prediction lead produced by the selected horizon.

All experiments were conducted under identical hardware configurations and respiratory waveforms, alternating between Mode A and Mode B testing to eliminate random errors.

## 3. Results

This section systematically presents comparative experimental results and analyzes them in terms of dynamic registration accuracy and respiratory gating response performance to validate the effectiveness of this integrated system.

First, during system validation, the design complexity of QR codes was confirmed as a key variable affecting the stability of HoloLens pose recognition. As summarized in Table 1, increasing encoding density significantly reduced pose jitter, while version differentiation played a critical role in preventing ID-level coordinate misassignment. The first round of experiments employed simplified QR codes. Their sparse features resulted in excessively high initial similarity between adjacent markers, triggering ambiguity in SLAM feature estimation. When two spatially adjacent QR codes entered the field of view simultaneously, pose estimation produced abnormal angular deviations exceeding 15°. This stemmed fundamentally from spatial constraint degradation caused by insufficient feature points.

Subsequent experiments upgraded to medium-complexity QR codes (6 × 6 same-version encoding). While increased information density mitigated initialization ambiguity, identical encoding versions rendered the ID recognition mechanism ineffective: during rapid motion or partial occlusion, the system erroneously assigned QR code 1’s spatial coordinates to marker 5 (coordinate misplacement rate as high as 37%), causing structural disruption in spatial mapping relationships.

The final solution employs fully differentiated QR codes (7 × 7 versions V1–V5 with independent encoding), achieving triple optimization through introducing unique semantic constraints in encoding. High-density feature points enhance adjacent marker differentiation, compressing pose estimation jitter to ±0.3 mm; version differences construct a discrete encoding space, reducing coordinate misalignment rates below 0.2%; Even under extreme viewing angles of 70° or 30% local occlusion, recognition success rates remained consistently above 98%.

This evolutionary path reveals that QR code complexity must co-evolve with encoding uniqueness. While sufficient information density ensures pose estimation accuracy, version differentiation is crucial for achieving ID-level spatial topology locking. This approach holds universal design value for scenarios with extremely low tolerance for error, such as surgical navigation.

**Figure 9:**
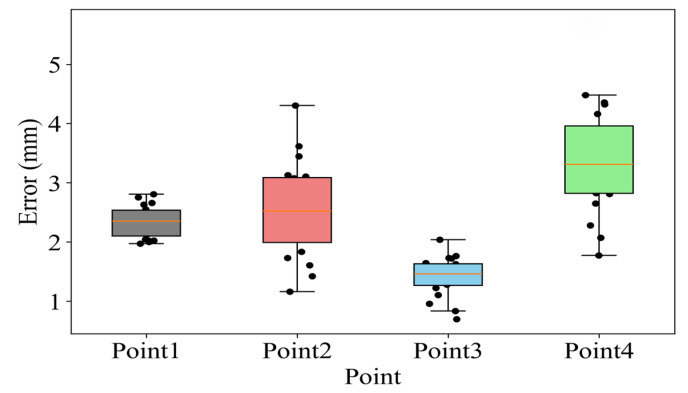
Three-Dimensional registration alignment error for Point 1 to Point 4. The orange line indicates the median value.

The 3D registration alignment error is shown in Figure 9, with an average alignment error of 2.15 ± 1.05 mm, a maximum value of 4.80 mm and a minimum value of 1.42 mm. The error of point 1 is 2.30 ± 0.22 mm, the error of point 2 is 2.42 ± 0.69 mm, the error of point 3 is 1.47 ± 0.35 mm, and the error of point 4 is 3.26 ± 1.04 mm. Point 1 and Point 3 are points closer to the CT coordinate origin, while Point 4 is a point farther from the CT coordinate origin.

**Figure 10:**
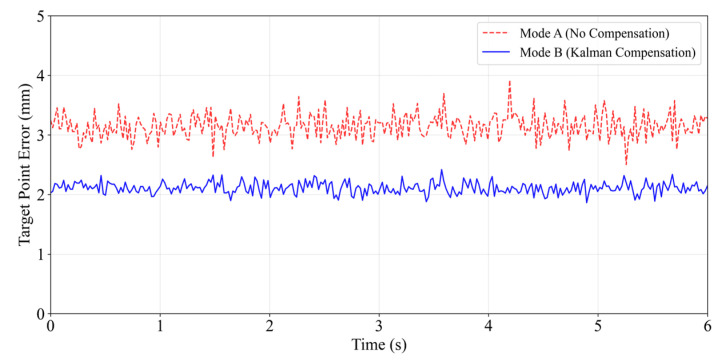
Dynamic Target Point Error Comparison Chart.

Analysis of dynamic target point errors clearly demonstrates the superiority of the Adaptive Kalman filter prediction compensation algorithm. Figure 10 visually illustrates the error variation between the MR virtual target point and the actual target point during a typical respiratory cycle under Mode A (no compensation) and Mode B (Kalman compensation). It can be observed that enabling compensation significantly reduces both the magnitude and fluctuation of the error.

The quantitative analysis results are summarized in Table 2. When tested with a standard sine wave, Mode A exhibited an average TPE of 2.82 ± 0.91 mm, whereas Mode B significantly reduced this to 2.24 ± 0.39 mm (*p* < 0.001, paired *t*-test). In the real breathing waveform test (steady breathing), Mode B also performed excellently, reducing the average TPE from 3.15 ± 1.23 mm to 2.11 ± 0.58 mm (*p* < 0.001), demonstrating the algorithm’s robustness to non-ideal breathing patterns.

**Figure 11:**
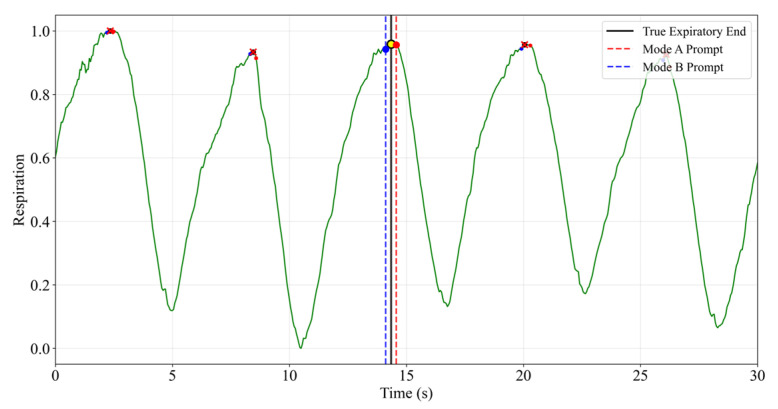
Respiratory Gating Timing Comparison Diagram. Cross symbols indicate the detected respiratory peaks. Colored dots mark the intersections between gating times and the respiratory waveform, with the yellow dot indicating $T_{gold}$ of the representative cycle and the red and blue dots indicating the prompt times of Mode A and Mode B, respectively.

The performance comparison results of the respiratory gating cueing system are shown in Figure 11 and Table 2. The time-series diagram in Figure 8 clearly demonstrates that cues based on Adaptive Kalman filter prediction (Mode B) are consistently delivered ahead of the actual end-inspiration point, whereas cues based on instantaneous detection (Mode A) exhibit significant delay.

Based on the quantitative data in Table 2, during the standard sine wave test: Mode A achieved a prompt accuracy of 100%, but its average prompt delay was 200 ± 31 ms, while Mode B achieved an average prompt time of −125 ± 45 ms, indicating a 125-ms advance in puncture recommendations while maintaining high accuracy. This trend persists in real respiratory wave testing, where Mode B achieves a lead time of −142 ± 52 ms with 92% accuracy. Experiments demonstrate that the Adaptive Kalman filter algorithm exhibits superior performance in predictive capabilities. However, for clinical application, it is necessary to further enhance the time delay compensation while maintaining a high level of accuracy.

Table 3 shows the performance comparison of the Adaptive Kalman filter under Mode B across three respiratory states. Based on the experimental results, the proposed algorithm demonstrated comparable accuracy in estimating respiratory signals during both Regular Breathing and Shallow Breathing states. However, its performance revealed limitations under irregular patterns. Specifically, the maximum absolute error increased to 4.62 mm during Shallow Breathing and further to 5.04 mm during Irregular Breathing. These elevated error margins indicate that the current model’s robustness is compromised when confronted with significant deviations from normal, periodic respiratory patterns, highlighting an important area for future refinement to enhance clinical reliability across diverse physiological conditions. The poor results of Irregular waveforms stem from high-frequency, large-amplitude random pulses and non-stationarity in the movement patterns.

## 4. Discussion

This study successfully integrates Adaptive Kalman filter-based respiratory motion prediction, mixed reality visualization, and intelligent gate-controlled prompts to construct and validate an innovative navigation system tailored for dynamic surgical environments. Experimental results consistently and robustly demonstrate that the system significantly enhances dynamic registration accuracy while intelligently and proactively guiding puncture timing.

Prior MR-assisted pulmonary intervention studies mainly focus on spatial visualization and lesion localization, often relying on breath-holding or static assumptions to mitigate respiratory motion. As summarized in recent reviews, respiratory-induced displacement remains a key limitation affecting MR accuracy, particularly for lower-lobe lesions. The present work differs by explicitly modeling respiratory dynamics and system latency, introducing a predictive, timing-aware prompting strategy that complements existing MR spatial navigation approaches.

The system’s core contribution lies in upgrading navigation from passive “real-time tracking” to active “proactive compensation” through Adaptive Kalman filtering. The approximately 50% improvement in dynamic registration accuracy is not only statistically significant but also clinically meaningful. It reduces errors from levels potentially affecting small nodule localization accuracy to a clinically acceptable range (generally considered less than 2–3 mm for most biopsy procedures) [26,27,28]. This precision gain directly translates to stable MR visualization, tight alignment between virtual models and actual organs, and greatly enhances physician confidence in navigation information.

More importantly, the respiratory gating alert system achieves a leap from informing the present to predicting the future. With an average advance warning of approximately 142 ms, it restores valuable preparation time to physicians, enabling them to calmly plan and execute puncture maneuvers. This is expected to reduce errors or multiple punctures caused by rushed operations, ultimately enhancing surgical safety and efficiency. Although the proposed predictive gating system successfully provides an advanced notification of the optimal puncture timing, preliminary in-house testing with team members has uncovered a critical challenge in translating technical performance into clinical practice: the variability in operator response time. Testing revealed significant variations in reaction time from receiving MR prompts to actually performing the puncture action among different individuals, and even among the same person in different situations.

This inherent human factor variability, coupled with any potential instability in system latency, could potentially lead to missed opportunities for puncture within the brief ideal window, thereby impacting the ultimate success of the procedure. This observation underscores that a truly robust dynamic navigation system must not only provide a theoretically optimal timing but must also integrate the operator into the entire control loop. Future work should focus on developing more adaptive interaction strategies. For instance, implementing a multi-stage alert [29] (e.g., Get Ready followed by Puncture Now) based on the remaining duration of the respiratory cycle and operator habits, or exploring semi-autonomous robotic assistance where the prompt signal translates into a constrained, active execution, could be promising directions [30,31]. Such advancements would minimize the dependency on human reaction latency and ensure the stable and reliable translation of system performance into clinical benefit.

Additionally, while it performs well under the steady breathing patterns observed in this study, its performance may deteriorate when handling coughing, hiccups, or highly irregular breathing patterns. Moving forward, we will explore more sophisticated models—such as predictive models incorporating deep learning [32]—to enhance robustness. Second, the current system relies on an external optical tracking system for respiratory signals, which may encounter occlusion and spatial constraints during clinical deployment. Integrating more portable respiratory monitoring sensors—such as abdominal belts or surface-mounted inertial measurement units [33]—will be a key direction for future clinical translation.

Furthermore, validation of this study is currently limited to physical phantoms. While phantom experiments provide valuable preliminary performance and feasibility data, factors such as the mechanical properties of biological tissue [34] and needle path deviation remain unaccounted for [35,36]. Consequently, animal studies will be an indispensable next step to evaluate the system’s overall efficacy in a more physiologically realistic environment. Finally, we will focus on optimizing the system’s overall latency and developing more sophisticated MR human–machine interfaces. These enhancements could include displaying confidence intervals for predicted targets or providing predictive information for multiple future respiratory cycles, thereby further improving the system’s practicality and reliability.

From a methodological perspective, the proposed system was verified using a respiratory motion simulator and physical phantoms to specifically evaluate its temporal prediction accuracy, dynamic registration stability, and prompt timing performance under controlled and repeatable conditions. This verification strategy was deliberately chosen to isolate respiratory motion and system latency effects from confounding variables such as operator skill, tissue deformation, and procedural variability.

By employing a simulator with known ground-truth respiratory phases and repeatable motion patterns, the validation focuses on the core technical contribution of this work—namely, predictive respiratory synchronization and proactive MR guidance—rather than downstream clinical outcomes. Such a staged validation approach is consistent with early-phase evaluation of navigation and guidance systems, where establishing algorithmic reliability and performance boundaries is a prerequisite for subsequent animal and clinical studies.

In summary, the experimental results presented in this study were obtained under controlled conditions using a respiratory motion simulator. While this method possesses core advantages such as repeatability, safety, and known benchmarks for verifiable algorithms, it represents only the first critical stage in the translation process. Subsequent logical steps include: (1) Conducting in vitro validation using human models incorporating tissue-mimetic materials to assess needle-tissue interaction effects; (2) In vivo animal studies to evaluate system performance in real-world settings; (3) Clinical pilot trials, which require further engineering optimization to meet clinical ergonomics requirements and obtain necessary ethical approvals. This study successfully establishes the technical feasibility and core performance benchmarks of the proposed MR navigation and gating system, laying a solid foundation for subsequent translational steps.

## Figures and Tables

**Table 1:** Quantitative comparison of registration accuracy under different QR code designs.

QR Configuration	Encoding Density	Translational Jitter (mm)	Angular Deviation (°)	ID Misassignment Rate (%)
Simplified QR	Low	2.6 ± 0.9	15.8 ± 3.2	35
6 × 6 QR (same version)	Medium	0.9 ± 0.4	4.3 ± 1.1	10
7 × 7 QR (V1–V5)	High	0.29 ± 0.08	1.2 ± 0.5	0

**Table 2:** System Performance Evaluation Results.

Test Conditions	Mode	Average TPE (mm)	Prompt Accuracy Rate (%)	Average Prompt Time (ms)
Standard sine wave	Mode A (No Compensation)	2.82 ± 0.91	100	200 ± 31
	Model B (Kalman Compensation)	2.24 ± 0.39	100	−125 ± 45
True Breathing Wave	Mode A (No Compensation)	3.15 ± 1.23	90	195 ± 38
	Model B (Kalman Compensation)	2.11 ± 0.58	92	−142 ± 52

**Table 3:** System Performance Evaluation Results.

Breathing State	Average TPE (mm)	Maximum Error (mm)	Prompt Accuracy Rate (%)	Average Prompt Time (ms)
Regular Breathing	2.11 ± 0.58	2.84	92	−142 ± 52
Shallow Breathing	2.23 ± 0.86	4.62	91	−142 ± 63
Irregular Breathing	3.36 ± 0.76	5.04	85	−210 ± 83

## Data Availability

The data used in this study are publicly available from the GitHub repository: https://github.com/SangWoonJeong/Respiratory-prediction, associated with Jeong et al. [21], and were accessed on 15 July 2025. The version of the software corresponds to the publicly available repository state at the time of access.

## Appendix A. Air Tightness and Stability Testing

To ensure the reliability and repeatability of the respiratory simulation, the assembled pneumatic platform underwent rigorous testing for air-tightness and motion stability. Under confirmed airtight conditions, the platform was driven by a standard sinusoidal waveform (amplitude: 10 mm, period: 4 s). A high-precision optical motion capture system was employed to continuously monitor and record the 3D trajectory of a marker located at the center of the dynamic region of the airbag over more than 50 respiratory cycles. The cyclic repeatability of the platform was assessed by analyzing the position of this marker at the end-inspiration phase for each cycle. Results demonstrated that the standard deviation of the 3D positions of all end-inspiration points was less than 0.3 mm, with the maximum positional fluctuation tightly constrained within 1.0 mm. This confirms that the simulation platform is capable of generating highly stable and repeatable dynamic displacements, thereby providing a dependable foundation for the subsequent performance evaluation of the navigation system.
